# Plate configuration in midshaft clavicle fractures: influence of working length, screw number, and plate length—a retrospective cohort study of 355 patients

**DOI:** 10.1007/s00590-026-04728-8

**Published:** 2026-03-30

**Authors:** Michelle Antonia Hinkelmann, Desiree Schibler, Flurina Frei, Hans-Christoph Pape, Florin Allemann, Christian Hierholzer, Christiane Barthel

**Affiliations:** 1 Traumatology, University Hospital of Zurich, Zurich, Switzerland; 2 Shoulder and Elbow Surgery, Schulthess-Klinik, Zurich, Switzerland; 3 Traumatology, University Hospital of Zurich, Zurich, Switzerland

**Keywords:** Clavicle fracture, Midshaft clavicle, Plate osteosynthesis, Plate working length, Screw configuration, Plate length, Mechanical implant failure, Callus formation

## Abstract

**Introduction:**

Midshaft clavicle fractures are commonly treated with plate fixation; however, the selected plate working length varies widely. Biomechanical principles suggest potential effects on fracture healing, but clinical evidence remains limited, and the roles of screw number and plate length are not well defined. This study evaluated whether plate working length, screw number, and plate length influence callus formation, complications, or implant removal in operatively treated diaphyseal clavicle fractures.

**Methods:**

This retrospective single-center study included patients aged ≥ 16 years treated with plate fixation for diaphyseal clavicle fractures between 2016 and 2024. Implant configuration and clinical outcomes were extracted from the institutional database. Patients were grouped by the number of free plate holes adjacent to the fracture (0, 1, 2, or ≥ 3). Radiographic healing was assessed by three independent observers. Statistical analyses included chi-square tests, ANOVA or Kruskal–Wallis tests depending on data distribution.

**Results:**

Among 355 patients, plate working length showed no association with callus formation, complications, or implant removal. Constructs with mechanical implant failure contained fewer screws on average than those without failure (6.43 vs. 7.14 screws), although the number of failure events was small. Plate length showed no significant relationship with callus formation or complications.

**Conclusion:**

Plate working length showed no meaningful association with healing or complication rates. Constructs with mechanical implant failure contained fewer screws on average, suggesting that secure screw fixation may be more relevant for construct performance than modifications of working length or plate length. Plate length itself did not significantly influence outcomes.

**Level of evidence:**

Level III, retrospective cohort study.

## Introduction

Midshaft clavicle fractures account for approximately 80% of all clavicle fractures, with an incidence of 29–64 per 100,000 population per year [1, 2]. Displaced fractures are increasingly treated with plate osteosynthesis, achieving union rates exceeding 95% [3–6]. However, substantial variation exists in implant configuration, particularly regarding working length—a construct parameter describing the span between screws adjacent to the fracture. In long bone fractures, biomechanical principles suggest that longer working lengths promote callus formation through controlled interfragmentary motion and reduce stress concentration by distributing forces over a greater plate segment [7, 8]. These advantages might benefit healing of clavicle fractures subjected to complex multiplanar loading [9, 10]. However, clinical evidence linking working length to outcomes in clavicle fractures is sparse [11, 12].

Against this background, it remains unclear whether these biomechanical adaptations translate into clinically relevant differences in healing, complications, or the high hardware removal rate of 20–50% [13, 14]. Moreover, the independent contributions of total screw number and overall plate length - two additional determinants of construct stiffness - have not been systematically evaluated.

This study analyzed operatively treated diaphyseal clavicle fractures to evaluate the relationship between plate working length and clinical outcomes, with secondary analysis of screw number and plate length. We hypothesized that longer working lengths would be associated with improved healing, enhanced callus formation, lower complication rates, reduced implant removal rates, and decreased refracture risk afterwards.

## Materials and methods

This retrospective single-center study included all patients treated for diaphyseal clavicle fractures at the level 1 trauma center between 2016 and 2024. Inclusion criteria were age ≥ 16 years, written informed consent for the scientific use of retrospective data in the form of a generalized consent, and operative treatment with plate fixation. Exclusion criteria were lack of consent, nonoperative management, or absence of postoperative follow-up.

A total of 355 patients met the eligibility criteria. Surgical details and implant configuration were extracted from the institutional clinical information system and categorized according to three construct variables of interest: plate working length, total screw number, and plate length. Plate working length was assessed radiographically and defined as the number of unused plate holes adjacent to the fracture site (0, 1, 2, or ≥ 3). In biomechanical studies, working length describes the distance between the innermost screws on either side of the fracture. Because this distance could not be measured directly in this retrospective radiographic analysis, the number of unused plate holes was used as a surrogate measure of working length. In our standard 3.5-mm locking compression plates, the center-to-center distance between adjacent holes is approximately 12 mm, allowing an approximate estimation of the working span. Total screw number represented the overall number of screws placed in the plate, and plate length was measured as the total number of plate holes. Patients were stratified into four working-length groups based on the number of unused holes adjacent to the fracture (0, 1, 2, or ≥ 3).

Radiographic healing was evaluated using standard postoperative follow-up radiographs. Callus formation was defined as visible bridging callus on conventional two-plane follow-up radiographs. Callus formation was assessed independently by three observers using serial radiographs, with one observer repeating the assessment after three months to determine inter- and intra-observer reliability. Clinical outcomes included operation time, complications, and implant removal (IR). Refracture events following implant removal were also recorded.

All statistical analyses were performed using Python™. Inter- and intra-observer agreement for callus assessment was quantified using Cohen’s and Fleiss’ kappa statistics. Group comparisons used chi-square tests for categorical variables and one-way ANOVA or Kruskal–Wallis tests for continuous variables, depending on distribution. The association between total screw number and material failure was evaluated by comparing screw counts between failure and non-failure cases. Statistical significance was set at *p* < 0.05.

## Results

A total of 355 patients were included. The mean age was 43.3 years (*median* = 41 years; *range* = 17–90 years). Most patients were men (290; 81.7%). Fractures involved the left side in 184 cases (51.8%) and the right side in 171 cases (48.2%). The overall complication rate was 6.2% (22 cases). Inter-rater agreement for callus assessment was moderate (Fleiss’ κ = 0.48). Intra-observer reliability was also moderate, with a Cohen’s κ of 0.45 for repeated assessments.

**Fig. 1:**
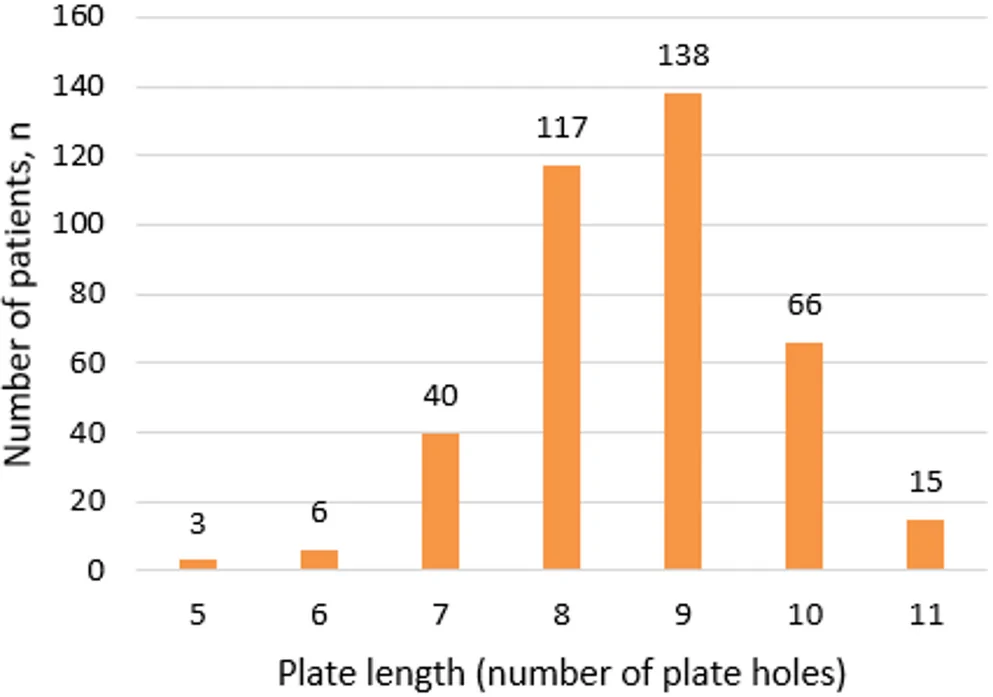
Distribution of patients according to plate length

**Fig. 2:**
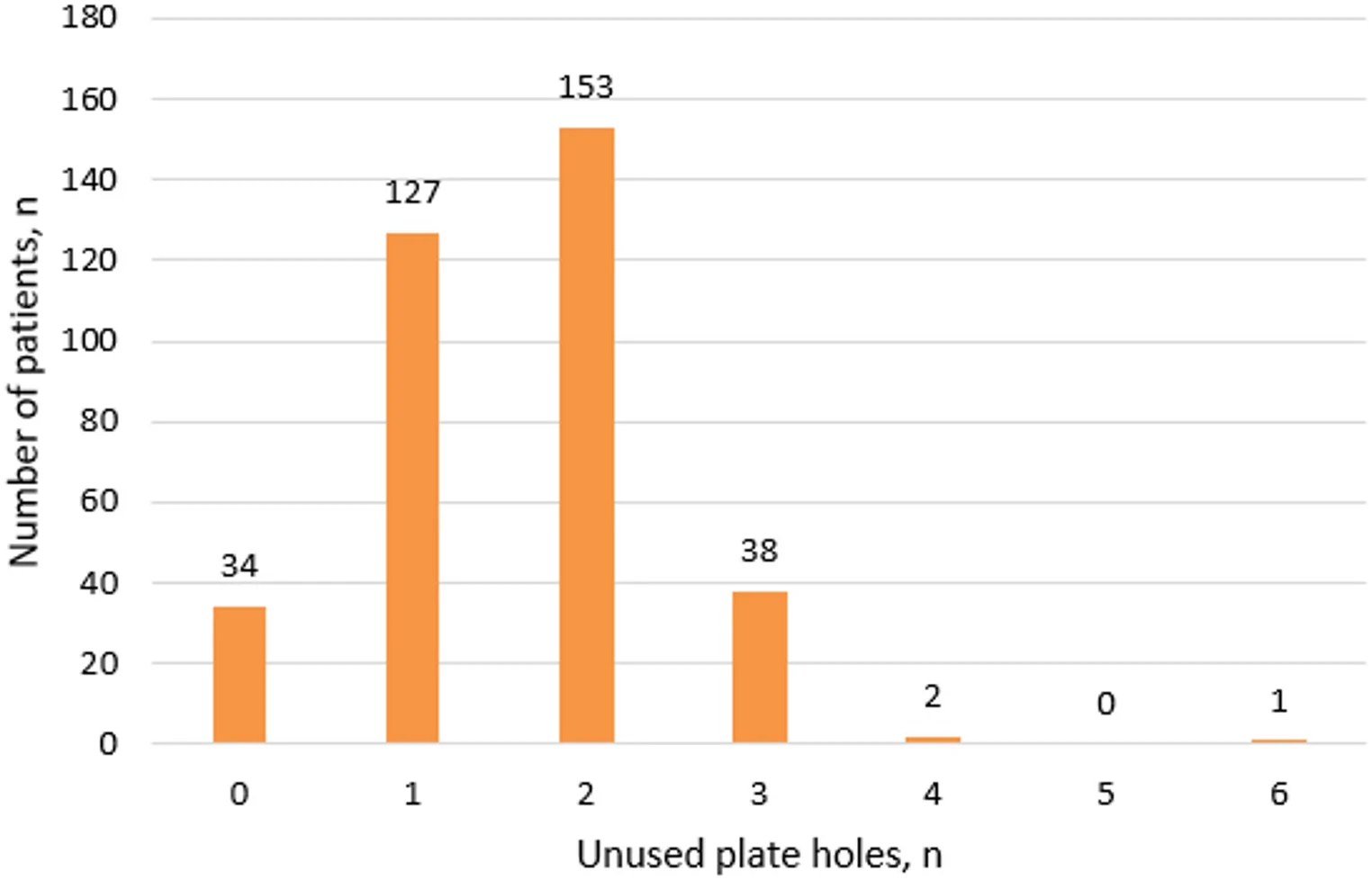
Distribution of unused plate holes adjacent to the fracture zone

Surgeons most commonly left one or two free holes adjacent to the fracture (one hole: 35.8%; two holes: 43.1%). Three free holes were used in 38 cases (10.7%), whereas constructs with zero free holes (9.6%) or ≥ four free holes (≤ 0.9%) were rare (Fig. 1, Table 1). Locking compression plates with seven to ten holes were used most frequently, with seven-hole plates accounting for 37 cases (10.4%), eight-hole plates for 112 (31.5%), nine-hole plates for 124 (34.9%), and ten-hole plates for 59 cases (16.6%). Very short plates (five or six holes) and very long plates (eleven holes) were uncommon (≤ 3.9%) (Fig. 2, Table 1). The total number of screws ranged from four to twelve, with most constructs containing six to eight screws. .

### Plate working length

Patients were grouped by the number of free holes adjacent to the fracture (0, 1, 2, or ≥ 3). Age (*p* = 0.168) and sex (*p* = 0.057) did not differ between groups. Fragmentation patterns differed significantly (*p* = 4.6 × 10^−6^), with more comminuted fractures treated with more free holes.

Operation time differed between groups (*p* = 0.0145). Procedures with ≥ three free holes had the longest mean duration (97.1 min vs. 82.0–90.6 min).

Callus formation did not differ by working length (*p* = 0.321). Among patients forming callus, time to callus was also similar (*p* = 0.185), with median values of 12 weeks in all groups except the zero-hole group (*median* = 6 weeks).

Complication rates were comparable (4.7–8.8%; *p* = 0.802). Implant removal occurred in 32% to 47% of patients without significant group differences (*p* = 0.142). Time to removal differed between groups (19.8 vs. 26.4 months; *p* = 0.047). Removal duration (*p* = 0.128) and refracture rates (0–20%; *p* = 0.189) showed no significant differences. (Table 2)

### Number of screws per plate

The total number of screws was associated with material failure. Patients with mechanical implant failure had fewer screws on average than those without failure (6.43 vs. 7.14 screws, *p* = 0.0127).

Screw number showed no association with callus formation (*p* = 0.349), nonunion (*p* = 0.27), or implant removal (*p* = 0.981). Except for isolated material failure, screw number did not influence outcomes. (Table 3)

### Plate length

Plate length ranged from five to eleven holes. Complication rates (0–10%) showed no association with plate length (*p* = 0.193). Callus formation (71–100%) did not differ between groups (*p* = 0.407). Implant removal ranged from 26% to 67% with a nonsignificant trend toward higher removal in very short plates (*p* = 0.078). Overall, plate length did not significantly influence complications, callus formation, or implant removal. (Table 4)

## Discussion

This retrospective study of 355 operatively treated midshaft clavicle fractures examined the influence of plate configuration on radiographic and clinical outcomes.

### Working length and callus formation

Contrary to our initial hypothesis, plate working length, defined by the number of free holes adjacent to the fracture, did not affect callus formation, complications, or implant removal. Constructs with mechanical failure contained fewer screws on average, although the small number of failure events limits the statistical precision of this observation. Plate length, representing the total number of plate holes, showed no meaningful relationship with any outcome parameters. These findings suggest that construct behaviour in plating of diaphyseal clavicle fractures may be influenced less by working length or plate length than by secure screw purchase.

We found no association between plate working length and callus formation (*p* = 0.321), time to callus formation (*p* = 0.185), or complications (*p* = 0.802). This contrasts with biomechanical principles derived from long-bone fractures, where increased working length reduces construct stiffness and promotes interfragmentary micromotion and thus callus formation [7, 8, 15]. Several factors likely explain the absence of such effects in the clavicle. First, the absolute working length in clavicle plating is small; even constructs with three free holes span only 9–12 mm, which may be insufficient to meaningfully alter stiffness [7, 16]. Second, the clavicle’s S-shaped geometry, thin cortices, and multiplanar loading create a distinct mechanical environment in which local screw purchase may dominate construct behaviour [9, 17].

### Working length and fracture morphology

Working length also correlated strongly with fracture morphology: more comminuted fractures were treated with more free holes (*p* = 4.6 × 10^−6^), indicating that working length primarily reflected fracture complexity rather than surgeon preference. Because of this association, additional multivariable analyses adjusting for fracture fragmentation and age were explored. However, due to the very high callus rate and the low number of complication events, the models were statistically unstable, and the final analysis therefore remained descriptive. Despite these differences, outcomes remained comparable across groups, suggesting appropriate adaptation to fracture severity. Time to implant removal differed modestly between groups (19.8 vs. 26.4 months; *p* = 0.047), while overall removal rates remained similar (32–47%, *p* = 0.142). This likely reflects prolonged rehabilitation in more complex fractures [18] as implant removal in clavicle fractures is typically driven by soft-tissue irritation rather than mechanical factors [18, 19].

### Screw number and material failure

Cases with mechanical implant failure had fewer screws on average than those without failure (6.43 vs. 7.14 screws). However, the small number of failure events limits the statistical precision of this observation. This finding is consistent with biomechanical data showing that increased screw purchase improves load distribution and reduces stresses at the plate–bone interface [20, 21]. Adequate screw fixation is particularly important in comminuted fractures, where individual fragments provide limited cortical support [21, 22]. Pseudoarthrosis demonstrated the opposite numerical pattern, occurring in cases with higher screw counts (8.0 screws), although not reaching statistical significance (*p* = 0.27). This trend may reflect surgeon selection of more robust constructs for multifragmentary higher-risk fracture patterns or a potential increase in construct stiffness that could limit interfragmentary motion [8, 15]. Notably, screw number showed no relationship with callus formation (*p* = 0.349) or implant removal rate (*p* = 0.981).

### Plate length and clinical outcomes

Plate length (five to eleven holes) was not associated with complications (*p* = 0.193), callus formation (*p* = 0.407), or implant removal (*p* = 0.078). This contrasts with prior reports suggesting increased removal rates with longer plates [13], likely reflecting methodological differences. That study included non-anatomic implants with higher profiles, whereas our cohort consisted primarily of precontoured locking plates with reduced prominence [19, 23]. Soft-tissue irritation, the main driver of hardware removal in clavicle osteosynthesis, is strongly influenced by implant profile and position rather than hole count [17, 18]. The trend toward higher removal rates in very short plates may relate to inadequate screw purchase and increased terminal stress - phenomena demonstrated in biomechanical models [17] - though the clinical impact in this cohort appears minimal when appropriate fixation is achieved.

### Clavicle-specific considerations

The clavicle’s unique anatomy helps explain why neither working length nor plate length substantially influenced outcomes. Its thin cortices and variable cross-sectional geometry constrain screw trajectories, making secure screw purchase a dominant factor in construct stability [21, 24]. Plate position also affects mechanical behaviour: where superior plating provides higher bending resistance, whereas anteroinferior plating reduces prominence but can alter stiffness [17, 23]. The clavicle’s subcutaneous location means that symptomatic hardware is driven largely by soft-tissue irritation rather than load-sharing mechanics [18, 19].

### Clinical implications

Comminuted fractures present additional challenges. Free holes adjacent to the fracture can act as stress risers and therefore require careful balancing of working length and screw placement. In our study, surgeons appropriately applied longer constructs for multifragmentary patterns, resulting in comparable healing and complication rates across working-length groups. These findings suggest that in constructs with fewer than three bicortical screws on each side of the fracture, construct behaviour is governed more by screw quality and distribution than by working length or plate length.

### Limitations

This study has several limitations. Implant configuration was not randomised but reflected surgeon judgment, which may introduce selection bias. Working length was assessed using a radiographic surrogate based on unused plate holes and therefore does not capture the exact millimeter distance between screws. The study predominantly evaluated precontoured locking plates; thus, the findings may not be generalisable to other implant types.

Although the degree of fragmentation was included in the analysis, detailed fracture morphology—such as spiral, oblique, or transverse patterns—was not systematically classified, which may influence construct mechanics and fixation behaviour. In addition, locking compression plates were manually contoured intraoperatively, introducing variability in plate shape, material stress, and final implant position that could affect construct stiffness and local loading conditions.

Callus formation was assessed using plain radiographs, which may underestimate subtle differences detectable on advanced imaging. Patient-reported outcomes and functional measures were not routinely collected. Furthermore, the median follow-up duration may not capture late complications occurring beyond two to three years.

Finally, the exact position of the fracture within the clavicle shaft (medial, central, or lateral diaphyseal region) was not analysed separately. Fracture location within the shaft may influence plate positioning and the number of screws that can be placed on each side of the fracture, which could affect construct configuration.

## Conclusions

In this cohort of operatively treated midshaft clavicle fractures, plate working length showed no association with callus formation, complications, or implant removal. Constructs with mechanical implant failure contained fewer screws on average, suggesting that secure screw fixation may contribute to construct stability. Plate length demonstrated no meaningful relationship with healing, complications, or implant removal. These findings suggest that, in clavicle shaft fixation, secure screw purchase is more clinically relevant than modifications to plate working length or plate length.

**Table 1:** Overview of plate lengths and number of unused plate holes adjacent to the fracture

Category	*n*	%
*Plate length (total number of holes)*		
5 holes	3	0.80
6 holes	6	1.70
7 holes	37	10.40
8 holes	112	31.50
9 holes	124	34.90
10 holes	59	16.60
11 holes	14	3.90
*Number of free holes adjacent to the fracture*		
0 free holes	34	9.60
1 free hole	127	35.80
2 free holes	153	43.10
3 free holes	38	10.70
≥ 4 free holes	3	0.90

**Table 2:** Outcomes by plate working length

Outcome	Group differences	Key values	*p *value
Age	No difference	–	0.168
Sex	No difference	–	0.057
Fragmentation	More comminution → more free holes	–	**4.6 × 10** ^−6^
Operation time	Longest with ≥ 3 free holes	97.1 vs. 82.0–90.6 min	**0.0145**
Callus formation	No difference	–	0.321
Time to callus	No difference	Median 12 w (except 0 holes = 6 w)	0.185
Complications	No difference	4.7–8.8%	0.802
Implant removal rate	No difference	32–47%	0.142
Time to implant removal	Different between groups	19.8 vs. 26.4 mo	**0.047**
Removal duration	No difference	–	0.128
Refracture after IR	No difference	0–20%	0.189

The bold values indicate statistically significant results (p 0.05)

**Table 3:** Outcomes by screw number

Outcome	Comparison	Key values	*p *value
Material failure	Fewer screws in failures	6.43 vs. 7.14 screws	**0.0127**
Callus formation	No association	–	0.349
Nonunion	No significant difference	–	0.27
Implant removal	No association	–	0.981

The bold values indicate statistically significant results (p 0.05)

**Table 4:** Outcomes by plate length

Outcome	Comparison	Key values (%)	*p *value
Complications	No association	0–10	0.193
Callus formation	No difference	71–100	0.407
Implant removal	Trend toward higher removal in very short plates	26–67	0.078
Overall interpretation	No significant effect	–	–

## Data Availability

The datasets generated and analyzed during the current study are not publicly available due to institutional data protection regulations but are available from the corresponding author on reasonable request.
